# Perceptions of Health, Health Care and Community-Oriented Health Interventions in Poor Urban Communities of Kinshasa, Democratic Republic of Congo

**DOI:** 10.1371/journal.pone.0084314

**Published:** 2013-12-19

**Authors:** Vivi Maketa, Mimy Vuna, Sylvain Baloji, Symphorien Lubanza, David Hendrickx, Raquel Andrea Inocêncio da Luz, Marleen Boelaert, Pascal Lutumba

**Affiliations:** 1 Department of Tropical Medicine, University of Kinshasa, Kinshasa, Democratic Republic of Congo; 2 Epidemiology unit, Institut National de Recherche Biomédicale, Kinshasa, Democratic Republic of Congo; 3 Epidemiology unit, Institut Supérieur de Techniques Médicales, Kinshasa, Democratic Republic of Congo; 4 Department of Social Science, University of Kinshasa, Kinshasa, Democratic Republic of Congo; 5 Department of Public Health, Institute of Tropical Medicine, Antwerp, Belgium; 6 Telethon Institute for Child Health Research, The University of Western Australia, West Perth, Australia; 7 International Health Unit, Faculty of Medicine, University of Antwerp, Antwerp, Belgium; University of Stirling, United Kingdom

## Abstract

In Democratic Republic of Congo access to health care is limited because of many geographical and financial barriers, while quality of care is often low. Global health donors assist the country with a number of community-oriented interventions such as free distribution of bednets, antihelminthic drugs, vitamin A supplementation and vaccination campaigns, but uptake of these interventions is not always optimal. The aim of this study was to explore the perceptions of poor urban communities of the capital Kinshasa with regard to health issues in general as well as their experiences and expectations concerning facility-based health services and community-oriented health interventions. Applying an approach rooted in the grounded theory framework, focus group discussions were conducted in eight neighborhoods of poor urban areas in the city of Kinshasa in July 2011. Study participants were easily able to evoke the city’s major health problems, with the notable exceptions of malnutrition and HIV/AIDS. They perceive the high out-of-pocket cost of health services as the major obstacle when seeking access to quality care. Knowledge of ongoing community-oriented health interventions seems good. Still, while the study participants agree that those interventions are beneficial; their acceptability seems to be problematic. This is chiefly put down to a lack of information and government communication about the programs and their interventions. Furthermore, the study participants referred to rumors and the deterring effect of stories about alleged harmful consequences of those interventions. Along with improving the provision and quality of general health care, the government and international actors must improve their efforts in informing the communities about disease control programs, their rationale and benefit/risk ratio. Directly engaging community members in a dialogue might be beneficial in terms of improving acceptability and overall access to health services and interventions. Novel ways of reducing the high out-of-pocket expenditure should also be explored.

## Introduction

Global health continues to face an enormous gap between the status of technological innovation in health and its implementation in the poor communities of the developing world where they are needed the most [1]. Although several global health initiatives have tried to improve the delivery of health interventions, many essential tools such as vaccines, drugs and diagnostics still have a very low uptake, especially in Africa where utilization rates of health services remain low [1,2]. Health promotion theories recommend addressing issues of low uptake by applying an interaction model that encompasses the links between the individual, the community and the broader socio-economic and cultural context [3]. 

In this study we explored the perceptions of poor urban communities of Kinshasa (the capital city of the Democratic Republic of Congo) with regard to health and health care issues in general and some specific global health interventions that are delivered directly to communities. Their knowledge and perceptions on current health interventions, accessibility to health care, prioritization of health problems, and their perceptions regarding the national health system were investigated. We hope to provide useful insights for policy makers by highlighting strengths and weaknesses of current public health strategies, as well as aid in the articulation of new strategies for expanding access to Primary Health Care (PHC) in the poor urban communities of Kinshasa [3]. 

## Methods

### Background

The overall health status of the population of the Democratic Republic of Congo is poor, as is reflected in several of the country’s health indicators [4]. The maternal mortality ratio is estimated at 549 deaths per 100,000 live births, with an annual infant mortality rate of 75 per 1000 live births. Malnourishment is also considered to be an important public health issue, with the proportion of under-five children in DRC suffering from chronic malnutrition estimated at 46% [5]. Furthermore, severe infectious diseases such as viral hemorrhagic fever, cholera, meningitis, plague, monkeypox, measles and poliomyelitis are epidemic threats, while other infections such as human African trypanosomiasis, tuberculosis, Buruli ulcer and malaria are endemic in the country. HIV/AIDS is also present, affecting 4.5% of the population aged 15 to 49 years [5].

This study was part of a multi-country research program initiated by the World Health Organization’s Special Programme for Research and Training in Tropical Diseases (WHO/TDR) to document perspectives of poor African urban communities on health and health care. Our study employed qualitative research and data analysis methods that are rooted within the theoretical frameworks of phenomenology and grounded theory [6,7]. Central to these approaches is the notion of understanding a certain phenomenon from the perspective of those experiencing it. They adopt an inductive approach to research, one which is not hypothesis-based, but instead takes on a bottom-up exploratory stance from which a theory or hypothesis for further research may be derived.

This particular qualitative study was conducted in Kinshasa between the 11^th^ and the 31^st^ of July 2011, by means of a series of focus group discussions (FGDs) conducted with 16 groups of 12 persons who were recruited through faith-based organizations (FBOs) and community-based organizations (CBOs) of poor urban communities. These organizations provided us with a direct avenue through which to approach a wide range of potential participants. To minimize the influence of age, only adult participants (>25 years) were invited to participate. No additional demographic information was collected from the participants, as there was a concern this might have introduced a selection bias based on participant reluctance to provide such information. The average FGD lasted 45 minutes to an hour. 

### Study area and selection of study sites

Kinshasa is divided into 24 administrative zones of which 18 are classified as urban and 6 as semi-urban. For our study, 4 out of the 18 urban zones were randomly selected. These were Bandalungwa, Matete, Limete and Bumbu. Table 1 summarizes their socio-demographic characteristics. A total number of 44 neighborhoods were included in the four urban zones: 7 in Bandalungwa, 13 in Matete, 11 in Limete and 13 in Bumbu. The 4 urban zones are provided with health care services by means of ‘fixed’ health centres on the one hand, and a number of community-based health interventions on the other hand. Some of the programs implemented in recent years include the distribution of insecticide treated nets (ITN) to households, the mass distribution of vitamin A & mebendazole to under five year olds, poliomyelitis vaccination campaigns, programs for antenatal and preschool care, and health education activities on sanitation, AIDS and sexually transmitted infections (STI). 

**Table 1:** Demographic characteristics of the study sites.

	**Bandalungwa**	**Matete**	**Limete**	**Bumbu**
**Surface (km^2^)**	6.8	4.9	18.0	5.7
**Population in 2008**	147,288	233,741	152,163	335,856
**Population density (inhabitants per Km^2^**)	23,454	47,898	8,453	58,922
**Main economic activity**	Civil servants, petty trade, informal sector			
**Number of neighborhoods**	7	13	11	13
**Habitat**	Old houses in durable material	Old houses in durable material	New houses in durable material	New houses in durable material
**Health facilities**	2 Reference health centres, 42 health centres- only 15 are in service.	1 Reference health centre, 63 governmental (2) and non-governmental (61)	1 Reference hospital and 2 hospital centre, several private health centres (unknown number)	1 Public health centre, 3 private hospitals, 29 dispensaries
**Partners in health**	CEMUBAC, SANRU, ECC/BOM , ADECOM M.M	BDOM, M.M.N, M.M.B, CESVI, UNFPA	CEMUBAC, SANRU, ECC/BOM , ADECOM M.M	CEMUBAC, BMAS, CS LELO, CS SCIBE

Source : Annual reports 2009 of the District Health Offices of Bandalungwa, Limete, Bumbu and Matete

We conducted a household survey prior to the main qualitative study to document the socio-economic level of the 44 neighborhoods in the four selected urban zones. This socio-economic survey assessed 100 randomly selected households per neighborhood. A total of 4400 households were interviewed throughout the 4 zones. Random selection of households was based on a systematic sampling interval *k* that was determined by dividing the number of households in the neighborhood obtained from the civil registry by 100. A first household was randomly selected following the EPI vaccine coverage survey method [8] and by considering the neighborhood’s civil administration office as the central point of the neighborhood.

On the basis of these pilot data, we selected the 2 neighborhoods with the lowest socio-economic level for each of the 4 zones. There were no major differences between the 8 neighborhoods in terms of their socio-economic level. 

Two FGDs were organized in each of the 8 neighborhoods: one with female and one with male participants. In total, 16 FGDs were organized as shown in Table 2. The FGDs were held in Lingala, the predominant language in Kinshasa, and were recorded on audiotape.

**Table 2:** Characteristics of the FGD discussions.

**FGDN.**	Administrative zone	Community	Nature of the organization	N. of participants	Gender	FGD Codification
**1**	Matete	Sumbuka	NGO	12	Male	FGD1
**2**	Matete	Sumbuka	CBO	12	Female	FGD2
**3**	Matete	Totaka	NGO	12	Male	FGD3
**4**	Matete	Totaka	FBO	12	Female	FGD4
**5**	Limete	Mombele	FBO	12	Male	FGD5
**6**	Limete	Mombele	FBO	12	Female	FGD6
**7**	Limete	Mfulu M’vula	FBO	12	Male	FGD7
**8**	Limete	Mfulu M’vula	FBO	12	Female	FGD8
**9**	Bandalungwa	Lumumba	FBO	12	Male	FGD9
**10**	Bandalungwa	Lumumba	CBO	12	Female	FGD10
**11**	Bandalungwa	Lubudi	FBO	12	Male	FGD11
**12**	Bandalungwa	Lububi	FBO	12	Female	FGD12
**13**	Bumbu	Mbandaka	CBO	12	Male	FGD13
**14**	Bumbu	Mbandaka	CBO	12	Female	FGD14
**15**	Bumbu	Kwango	FBO	12	Male	FGD15
**16**	Bumbu	Kwango	CBO	12	Female	FGD16

N.B : CBO : community based organization, FBO : Faith based organization.

### Data analysis

Audiotapes were transcribed verbatim in Lingala, and later translated from Lingala to French by the research team’s social scientist. The translations were verified by at least one co-investigator, who compared the translation to the original Lingala text. The data analysis was conducted by means of the French transcripts. The analysis report was later translated into English. The qualitative data analysis software package Atlas Ti was used to perform data analysis. This software allows the organizing and analyzing of unstructured datasets by fragmenting and categorizing data whilst keeping a link with the source documents (FGD transcripts in this case). 

General themes were identified based on the FGD question guide (Table 3) and used to create an initial set of semantic categories (community prioritization of health issues, community access to the health system, community perception of the health system, the primary care facility, health interventions, community participation and perception of the community-oriented health interventions) and subcategories that allowed for a systematic coding of the transcripts. Finally, additional codes were added during the analysis process to accommodate for supplementary themes and information that arose from the transcripts (community expectations, community suggestions, trust regarding the health system and the delivery of interventions, health system access, health system: health staff perception, community prioritization of disease, health system intervention cost, health system intervention training). Throughout this paper, we will illustrate our findings by means of excerpts taken from the FGDs. Each quote will include a reference to the relevant FGD, details of which can be found in table 2. 

**Table 3:** Focus Group Discussion Guide.

A**. Information on Health and existing health Interventions**
1. What are the health issues that you encounter in your community?
***Probes***:
Are they threats to your health?
Are they the main issues in your community?
2. What health interventions exist in your community?
***Probes:***
How long have these interventions been in place in your community?
Who is carrying out these interventions?
3. How do you feel about these interventions?
***Probes:***
Community perception of Priority interventions
Awareness
Coverage
Affordability
Access
B. **Community Participation and Involvement**
4. How were these interventions introduced in this community?
***Probes:***
Community participation/involvement?
What are the decision making structures in this community?
Process of participation?
- Which community members are directly carrying out this intervention in your community?
- How were the community members selected to carry out these interventions?
C**. Community perception of MoH**
5. What support did the health system provide to your community
***Probes:***
Free drugs
Training
Access to health facilities
D. Benefits **and Challenges**
6. What are the benefits of these interventions to your community?
7. What are your perceptions of the work of the existing community members delivering the interventions?
8. What factors hinder the work of existing community-selected members
9. What challenges do you face as a community due to the implementation of these interventions?
E. General **Information**
10. What are the health facilities in this community?
***Probes:***
Availability of health care facilities and how they function in the community
Nearness to health facilities
11. How well do these health care facilities satisfy the needs of your community?

All the audiotapes were converted to a digital format and were archived on a secured computer hard drive at the Department of Tropical Medicine of the University of Kinshasa. All original Lingala transcripts and French translations are stored on the same hard drive. The principal investigator is the only person with direct access to the data, although other research team members can be given access at their request. All data sources will be stored for a minimum of 5 years. 

### Ethical considerations

Prior to starting the data collection, ethical approval for this study was provided by the World Health Organization (WHO) Research Ethics Review Committee in Geneva, Switzerland and the Ethical Committee of the Public Health School of the University of Kinshasa in Kinshasa, DRC. The submitted research protocol detailed the research process as described in this paper, from the selection of the included neighborhoods to the implementation of the FGDs. Participation in the study was entirely voluntary and based upon the participant signing a written informed consent form. 

## Results

### Health priorities according to the community

Both men and women had similar viewpoints about health problems in the community. They all seemed to agree that malaria and typhoid fever were the two main health issues “*Malaria and typhoid are always present…*FGD7”. Non-communicable diseases such as diabetes and high blood pressure were also often mentioned “*Mainly malaria, high blood pressure, typhoid fever and also diabetes*… FGD12”, “*There are many diseases, diabetes… high blood pressure because people are under a lot of stress*. FGD13” “…*There is also high blood pressure, a person can fall over just like that and become paralyzed*. FGD16”. The two latter quotes illustrate the increased importance of non-communicable chronic diseases from the perspective of poor urban Congolese. The role that environmental factors play in health and wellbeing was also identified as an issue. The most commonly mentioned problems in this regard were the limited access to clean drinking water and the high levels of pollution in the area "… *but in our settings, water and dirt: there is too much of that… typhoid and malaria, those diseases result from dirt…*FGD6” “*We live in a polluted environment; we don’t see what the governments*’ *contribution is in public health services in our country*. FGD11”. “*Things get tough in our neighborhoods, septic systems provoke many diseases, they are exposed to the full sun and their contents evaporate into the neighborhood*… FGD10”. Many other health problems were touched upon by both genders during the FGDs, but generally in less detail than those listed above. These miscellaneous health problems included tuberculosis, eye problems, diarrhea, intestinal worms, hemorrhoids, anemia, epilepsy, sexual transmitted infections and a lack of sexual education, which was perceived to lead to early pregnancies. “… *if you look at the infection rate, it’s due to sexuality, … if you go there … you will notice that there are no girls beyond 23 years old that have not given birth yet, from 16, 17, 18 years old, they have given birth more than their mothers*. FGD1”. Surprisingly, HIV/AIDS nor malnutrition was mentioned as a concern in any of the FGDs. 

### Knowledge and perceptions regarding existing community-oriented health interventions

Communities consider specific health commodities or services provided by community-oriented programs to households as “aid” (“*assistance*”). While women only mentioned the Ministry of Health (MoH), men also mentioned the WHO and specific countries, such as Japan and Belgium, as the providers of such forms of aid. None of our study participants referred to the name of any NGO or other organization when discussing community-oriented programs. 

Both female and male FGD participants acknowledged the immunization of children against poliomyelitis as a notable health intervention, which they mostly simply referred to as ‘the vaccine’. "… *It was the vaccine for children from 0 to 5 years*… FGD15". The annual mass distribution campaigns of vitamin A and mebendazole to under-five year old children also seem to be well known by the communities. Mass distribution of ITNs to households was acknowledged by all the discussion groups, whilst this distribution had been organized only once in Kinshasa by the MoH, three years prior to our study. A group of men also mentioned prenatal and preschool care as a health intervention “…*mothers who go to the hospital to have their child weighed get advice; they* [*the nurses*] *give them advice, and show them how to avoid diseases*… FGD1”.

All the focus group discussants seemed to agree that community-oriented health interventions were (in principle) beneficial either to their children or to themselves. But, paradoxically, they also highlighted several factors that underlie a general reluctance from the population to accept such interventions. They complain about a lack of communication between the MoH and the targeted communities. “*In The beginning, the population seemed reluctant to accept those interventions, the population is not well informed, there is always a new wave that occurs related to politics… until now, the government does not protect us, we don’t understand …*FGD6 ”. Some discussants from the male FGDs highlighted a particular lack of information regarding the purpose of the interventions, and indicated this as the main reason why communities were reluctant to engage with some health programs, as the population does not feel involved in their process. The incapacity of some community health workers to provide adequate answers to the community’s questions about certain interventions also does little to instill confidence. Community health workers seem no better informed about the origins or the process of the health interventions than the community. A group of male participants indicated that the community volunteers should receive better training and be more knowledgeable about the interventions they are involved in. “[*When discussing health workers*]*… They should be more trained, they can’t say where the drugs come from or if the drug they administer can provoke fever in the child… they just give the vaccine without informing us about its possible side effects…*FGD11”. The women participating in our study voiced more concerns about health volunteer’s skills and the adequacy of their actions “… (they) *come to give drugs to children, do they know the degree of disease? Because every 3 months they come to give drugs against worms. I think they should perform medical examinations first*. FGD2". 

Moreover, health interventions concerning the free distribution of commodities, such as ITNs and vaccines, were sometimes looked upon with suspicion. In some cases free distribution was considered as a label of bad quality, which reduced the acceptability of such interventions by the community. “…*several people didn’t use it … they argue that other people should use it first because they don’t know where the product came from. If people don’t die after using the product, only then are they reassured.* [*When discussing ITNs*] FGD6”, “*Here in Kinshasa, some people refused what was given for free*. FGD15” “…*others said they give them away for free because they are already expired*. FGD7”. "[*Concerning vaccination*] *we don’t know what the process was, and some people don’t want them for their children because it might be poison that is being given to the children*… FGD10". The distrust towards the ITN distribution program was strengthened when people received phone-calls from relatives or friends abroad, telling them that the ITNs were not suitable for use. “Relatives from Europe *and Angola called us, telling us to avoid using the nets because there were medicines inside*… FGD4”. “*[When discussing ITNs*]* some people with relatives in Europe were contacted and they were told by their family that there are bad things inside, that it is poisoned…. Others said that they don’t want to use it because they say it is bewitched, children might die if they sleep under it*… FGD9". An important factor that reinforced the resistance of people to certain health interventions is the perception that there are no channels to obtain further information about an intervention, or to follow up in case a problem arises after the visit of a community health worker. “*Some people said: my child had diarrhea after the vaccination while others said that children lost appetite after vaccination… After the last vaccination two persons told us their children became ill and had severe fever*. FGD14”. 

### Perceptions regarding access to health care

Both the female and male focus groups had a broadly similar perception in regards to access to health care. Discussants felt that, geographically speaking, health facilities were generally located close enough for the inhabitants to access them comfortably. However, many FGD participants complained about the unsafe and insalubrious conditions they encountered in those health facilities. Most importantly, our study participants highlighted how people living in poor urban communities’ continue to experience limited access to health care services because of financial constraints. This was put forward as the main barrier for access to health care, even at a primary health care level. People have the feeling that everything is focused on money in those facilities. Some groups mentioned that formal health care providers are ready to welcome community members and serve them, but only if they have enough money to pay for the service. Furthermore, our FGD discussants considered the quality of health care to be directly proportional to their own out-of-pocket expenditure. They feel that they are not able to access what they consider to be high quality health care because it is perceived to be too expensive. They therefore sense that they are obliged to fall back on lower standard health facilities. “*All these facilities don’t deliver health care in the same way; actually, there are different prices which are often too high for the community* FGD2”. Some discussants reported on how some health providers may require a pledge if they want to be treated but have no cash to pay any fees upfront. “*There are people who ask for a pledge… if you have a television, you give it as a pledge and only then will they treat people, now imagine a sick person who has no pledge to give*. FGD11”. Both women and men mentioned that a financial barrier had also emerged during the ITN mass distribution campaigns when some community health workers started asking the people to pay for a service that should have been provided free of charge. “*I gave 500 Congolese francs (CF*)*, they said that they would come with the nets, but they have not returned until this day…*FGD8". “… [*When discussing ITNs*] *they were asking money, 200CF each and 600CF if you wanted three…*FGD3". 

N.B: At the time of the FGDs nine hundred CF was the equivalent of 1 US dollar.

### Perceptions regarding the health system

Female and male study participants felt that the government should be more responsible and more sensitive when it comes to the health problems of the urban poor. Their sentiment seems to be that the function of the health system is not primarily to provide healthcare. Instead it seems to be perceived as a system intent on making a profit through the commodification, marketing and monetization of health issues, a sentiment that generates frustration. 

Issues concerning a lack of trust regarding the tools, skills, expertise and intentions of health workers and practitioners associated to the health system were also raised, although mainly by male FGD participants. “*There are microscopes that don’t work… and sometimes the drugs that they administer are not even adequate for the diseases…*FGD11”. “*They were about to kill an elder who was suffering from high blood pressure… here they can kill people, me, they injected me with globin*. FGD1” The general lack of trust with regard to these health workers is highlighted in the following quotes “[*when discussing the provision of aid*]… *and that assistance, they* (*the health workers*) *give it to their families… we heard that another country sent assistance and it got into their shops, when they sent corn, they gave it to their families, to sell it*… FGD5”, “*Give us this assistance, if you give it to the hospitals, they will sell it*. FGD1”, “*They can show on television that they gave drugs to the hospitals but when you need it in case of an emergency, you will not find anything and you’ll return home with empty hands*. FGD12”. 

## Discussion

The inhabitants of Kinshasa’s poor urban communities seem to be well aware of some of the major health problems they are exposed to, with the notable exception of HIV/AIDS and malnutrition. The omission of HIV/AIDS in the discussions might be due to the sensitivity of the subject matter on the one hand, and Kinshasa’s relatively low infection rate of 1.9% in its sexually active population (15 to 49 years) on the other [5]. Strikingly, the lack of sexual education was only mentioned by one group of men, although teenage pregnancy is more than ever an issue in the poor urban population of Kinshasa [9]. Malnourishment remains a major health problem in Kinshasa, a city where the rate of chronic malnutrition is 19.1% and 15% in women of childbearing age and in under five year old children respectively [5]. The fact that our study participants did not mention it spontaneously as an important health problem was unexpected and is possibly related to the notion that malnutrition and starvation are considered more of an economic problem than a purely health-related one.

Even though the focus group discussions were stratified for gender, the perceptions of women and men about health issues and their prioritization were generally similar. This is likely to be a reflection of how health-related topics are commonly discussed within families and throughout the community, which may facilitate the sharing of knowledge and the cultivation of similar perceptions in regards to health and health service delivery. Furthermore, the fact that an infectious disease such as malaria is considered as a major threat by the community should not surprise in the context of a disease-endemic city where over 80% of hospitalizations are due to such infections and where the prevalence of fever in children, which is mostly related to malaria, is over 30% [5].

Both men and woman were aware of most of the health interventions that are currently being delivered in Kinshasa under internationally supported community-based programs. Only one group of men mentioned the antenatal care and pre-school care programs, which provide routine vaccinations and health checks to under-five year olds. Although the proportion of mothers making use of these programs reaches up to 85% in Kinshasa [5], none of the female FGDs mentioned them. This might be due to the fact that these services lack visibility, as they are provided through health facilities and are not delivered to communities through specific outreach campaigns, as is the case for better-known programs that are run in the area. 

Our study participants considered community-oriented health interventions as beneficial while they were generally reluctant to accept them. This apparent paradox might be related to the fact that the population is not provided with enough information about the aims, the origins and the distribution channels of those interventions. Health information messages are usually disseminated prior to the interventions through local television and radio stations. However, as there is a structural shortage of electricity in Kinshasa, especially in the city’s poor urban neighborhoods, those messages may rarely reach the targeted population. Therefore, other communication and information dissemination approaches need to be considered. The widespread availability of mobile phones throughout Kinshasa’s population provides opportunities for telephone text message and social media based communication strategies. More traditional outreach campaigns through the use of community health workers or using the channels provided by existing community organizations such as FBOs and CBOs might also lead to better informed communities and improved coverage of health delivery programs. Community participatory approaches, which involve the targeted communities in the process of planning and implementation of programs, have shown to significantly improve the access of rural African communities to essential health interventions [10]. Other studies have highlighted how such community participatory strategies lead to improved effectiveness of interventions [11-13]. Furthermore, involving communities in the intervention planning and implementation process is more likely to ensure that such public health strategies gain community support, elicit local action and foster feelings of community ownership [14]. Given the potential of such community participatory approaches for public health interventions, more research on effective and integrated community-centered delivery strategies for health programmes is called for [11,15,16]. A key factor in such a process is to verify that the proposed intervention addresses a health issue that is acknowledged and considered relevant by the community [12]. It is therefore important to improve overall understanding of community demand regarding health-related services and of their attitudes and practices towards certain health issues.

None of our FGD participants referred to any particular NGOs or other organizations when discussing the provision of community-oriented health programs. The only actors perceived to be playing a role in the implementation of health interventions were the Ministry of Health, the World Health Organization, and two countries: Japan and Belgium. No reference was made to any of the agencies operating within the United Nations system (UNDP, UNICEF and WFP), nor was any reference made to bilateral or multilateral aid providers (e.g. the World Bank, the European Union or specific countries, with the exception of Belgium and Japan). This finding strongly suggests that a significant proportion of inhabitants of Kinshasa’s poorer areas are not at all familiar with the organizations that actually implement many of the community-oriented health programs discussed in this paper. 

Moreover, what poor urban communities seem to perceive as an overall lack of transparency and communication leads to a general lack of trust in the health authorities, health care providers and the channels through which health interventions are provided. Those feelings of distrust are fuelled by rumors circulating in the population and are sometimes reinforced by messages from relatives abroad. Furthermore the lack of skills and knowledge of some health workers leads to a perception of amateurism. Consequently people may consider interventions to be unreliable, which leads to distrust. It is therefore not surprising that Kinshasa’s poor urban communities seem to be suspicious and reluctant when it comes to free health interventions, even if they recognize the potential of such programmes to improve health status.

This issue of trust in the frame of health care provision is complex, as it implies much more than person-to-person relationships and individual experiences of trust. Trust, or the lack thereof, is derived largely from societal and institutional foundations and the assumptions and expectations that individual actors ascribe to them [17]. Addressing issues of trust as they relate to health interventions in settings such as poor urban Kinshasa is therefore a challenging undertaking. It is nevertheless an important condition to ensure the effective implementation of such interventions, as illustrated by the examples provided in this study. Some argue that the rise of vertical, donor-driven health programs has failed to incorporate the notion of population trust into its frameworks, which primarily remain driven by mechanistic intervention strategies and quantity assurance mechanisms (as opposed to quality assurance mechanisms) [18]. Indeed, the dominance of such strategies might help explain why our study participants were not familiar with any of the organizations that were implementing health interventions at the time. A lack of communication and participation may therefore not only lead to a lack of trust, it may also lead to a lack of visibility and community recognition.

As we recruited our FGD participants through existing faith and community-based groups, we acknowledge that the outcomes presented in this paper are not necessarily generalizable to the larger communities our study participants belong to. The selection bias introduced through our sampling process might have affected the degree to which some topics were discussed in more detail than others, or were not discussed at all. However, given the diversity of the groups through which we recruited our participants and the large number of men and woman that took part in our FGDs, we feel confident that the issues reported here are highly relevant and merit the consideration of actors that are involved in the provision of health services and programs to the poor urban neighborhoods of Kinshasa.

Developing more efficient forms of health communication, providing health workers with better training, and involving the targeted communities during the development and implementation of interventions might improve their overall acceptance, especially in poor urban communities of Kinshasa. Policy makers should more actively engage the communities in each step of the intervention process and should track acceptance of health interventions over time if they hope to achieve sustainable outcomes [19].

DRC’s health system has been weakened by several factors, such as wars and the protracted political instability in the country [20]. Although the geographical accessibility of health services was not felt as a major concern in the urban areas in Kinshasa, the financial accessibility and the quality of care clearly was, as is also the case in other poor cities of Africa [15,21]. These problems lead to frustration and reinforce the feeling that the local government does not care about the health of the poor. In order to improve community access to good quality care, authorities should develop leadership, networks, capacities, and partnerships in a concerted effort to eliminate health inequities and provide quality health care for all [22]. Such political commitment may reduce the reluctance and skepticism of communities against the government’s interventions in the health sector. It might provide a better basis for implementing community directed interventions in the context of poor urban communities in Kinshasa. This might also be valid in other urban contexts, both within and outside of Africa, as such approaches have improved the access to health care in similar settings [10]. 

In conclusion, people living in Kinshasa’s poor urban communities seem to be aware of most of the area’s major health problems, with the notable exceptions of malnutrition and HIV/AIDS. Knowledge of ongoing disease-specific interventions in the city also seems to be good. Still, while the study participants agree that those health interventions are helpful; their acceptability seems to be problematic in some cases. This is chiefly put down to what communities experience as a lack of information and government communication about the programs and their interventions. Communities also perceive the high out-of-pocket cost of health services as a major obstacle when seeking access to good quality health services and are aware of the major inequities in health care provision. Novel ways of reducing the high community out-of-pocket expenditure should urgently be explored.

Along with improving the supply of health care in an overall effort to reduce health inequity, government and international actors must ensure communities are truly informed about health care programs, their rationale and their risk/benefits. Directly engaging community members in a dialogue seems the best way to improve acceptability and overall access to health services.
